# Complications in the treatment of periprosthetic joint infection of the hip: when do they occur?

**DOI:** 10.5194/jbji-6-295-2021

**Published:** 2021-07-29

**Authors:** Katherine Li, Mario Cuadra, Gregory Scarola, Susan Odum, Jesse Otero, William Griffin, Bryan D. Springer

**Affiliations:** 1 Department of Orthopaedic Surgery, Atrium Health Musculoskeletal Institute, 1025 Morehead Medical Dr., Suite 300, Charlotte, NC 28203, USA; 2 Department of Orthopaedic Surgery, Atrium Health Musculoskeletal Institute, 1320 Scott Ave., Charlotte, NC 28204, USA; 3 OrthoCarolina Research Institute, Inc., 2001 Vail Ave., Suite 300, Charlotte, NC 28207, USA; 4 OrthoCarolina Hip and Knee Center, 2001 Vail Ave. Suite 200A, Charlotte, NC 28207, USA

## Abstract

Prosthetic joint infection (PJI) is a devastating complication after total hip arthroplasty (THA). The common treatment in the USA is a two-stage exchange which can be associated with significant morbidity and mortality. The purpose of this study was to analyze complications in the treatment course of patients undergoing two-stage exchange for PJI THA and determine when they occur. **Methods:** We analyzed all patients that underwent two-stage exchange arthroplasty for treatment of PJI after THA from January 2005 to January 2018 at a single institution. Complications were categorized as medical or surgical and divided into interstage and post-reimplantation. Minimum follow-up was 1 year. Success was based on the MusculoSkeletal Infection Society (MSIS) definition. **Results**: 205 hips (203 patients) underwent first stage of planned two-stage exchange. The median age was 68 (interquartile range (IQR) 18). There were 97 males and 106 females. Overall, 73/205 (38 %) patients had at least one complication during treatment: 13.5 % (25/185) of patients experienced a medical complication and 28.1 % (52/185) a surgical complication; 2.4 % died within 1 year of surgery, and 4.9 % (15/203) had mortality at a median of 2.5 years (IQR 4.9); 27 % of patients had complications during the interstage period, most commonly being recurrence of infection requiring additional surgery (63 %); and 14 % of patients experienced a complication following reimplantation, most commonly persistence or recurrence of infection (59 %). While 92 % of patients that initiated treatment were ultimately reimplanted, only 69 % were infection free at 1 year and required no additional treatment. **Conclusions**: While two-stage exchanges for PJI in THA have been reported as successful, there are few reports of the complications during the process. In our series, significant numbers of patients experienced complications, often during the interstage period, highlighting the morbidity of this method of treatment.

## 1 Introduction

Periprosthetic joint infection (PJI) is a devastating and costly complication that occurs after total hip arthroplasty (THA). While efforts to minimize infection in primary THA have kept the rates between 0.8 % and 2 %, data show that the incidence may be on the rise (Springer et al., 2017). Unfortunately, coupled with the projected increase in number of THA procedures to be performed, there is a high likelihood that the absolute number of PJI cases will continue to increase (Kamath et al., 2015; Perfetti et al., 2017). The management of PJI will continue to be one of the costliest expenditures for payors in the coming years (Kurtz et al., 2012).

The most common treatment for chronic PJI following THA in the United States remains the two-stage exchange arthroplasty (Parvisi et al., 2010). During this process, the first stage involves removal of the implants, infected tissue, and debris and placement of an antibiotic spacer impregnated with high-dose antibiotics. This is done in conjunction with a course of intravenous antibiotic therapy followed by a period of observation (antibiotic holiday) for a variable amount of time to ensure infection eradication. Provided that the infection is deemed to be sufficiently treated, the patient then undergoes the second operation to reimplant the definitive prosthesis. The reported success of two-stage revision for hip PJI in the literature varies from 52 %–78 % (McPherson et al., 2002; Lim et al., 2009; Waagsbø et al., 2009; Kandel et al., 2019). In addition to the operative technique and antibiotic treatment, the success of this procedure is also dependent on factors outside of just the hip, including medical comorbidities, nutritional status, and health of the host (Berend et al., 2013; Brown et al., 2018; Khan et al., 2019).

The majority of the data available on two-stage exchange arthroplasty defines success only following reimplantation and often ignores complications that can occur during the treatment process. In order to fairly assess the outcomes of this treatment, all the patients submitted to the first stage of the revision process should be included in the study and not just the cases that achieved the second revision stage with reimplantation. This requires reporting on those that failed to reach the final goal (reimplantation). Defining not just the complications that occurred in the two-stage process but also when they occurred within the treatment time helps us to identify specific areas of improvement that could be targeted to decrease the overall rate of complications. For example, it may help to determine if there are cases where a one-stage procedure may be more appropriate if a particular comorbidity lends to a high complication rate during the interstage period. It may also assist in recognizing commonalities between the timing of the complications and lead to system changes involving the two-stage protocol. In an effort to minimize the variability in reporting the success or failures following treatment of PJI of the hip and knee, the MusculoSkeletal Infection Society (MSIS) developed a set of guidelines to standardize the definition of success in outcomes of treatment of PJI. The guidelines use a series of tiers to stratify success or failure and provide for a standardized level of reporting of overall treatment results (Fillingham et al., 2019).

The purpose of this study was to assess the overall outcomes of all patients (intention to treat) undergoing a two-stage exchange arthroplasty for treatment of chronic PJI in THA and define when complications occur during a two-stage exchange process.

## 2 Methods

**Figure 1:**
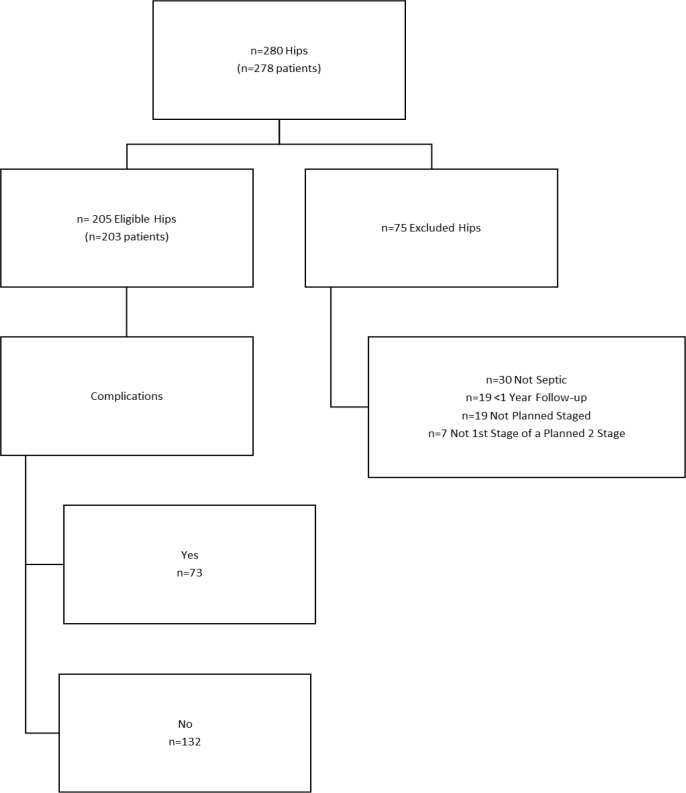
CONSORT (Consolidated Standards of Reporting Trials) flow diagram of eligible patients for two-stage exchange arthroplasty for chronically infected total hip arthroplasty (THA). All deceased patients and those with a retained spacer are included.

The institutional administrative database was interrogated to obtain a list of patients who underwent two-stage exchange arthroplasty for a diagnosis of PJI (280 hips in 278 patients) between January 2005 and January 2018 at our institution. Revisions performed outside our institution and those referred with a spacer in place from other institutions were excluded. A total of 75 patients did not meet the eligibility criteria and were excluded for either non-septic reasons or septic procedures exclusive of the first stage of a two-stage exchange (Fig. 1). Current procedural terminology codes 27091, in addition to a manual review of all septic and aseptic revisions, were used to identify those who underwent the first stage of a planned two-stage exchange arthroplasty for deep prosthetic infection. The final study sample included 205 hips in 203 patients with a minimum 1-year follow-up. Every patient met the definition of deep periprosthetic joint infection based on the criteria put forth by the modified definition of the MusculoSkeletal Infection Society (MSIS) based on a retrospective manual chart review and included either a sinus tract communicating directly with the joint or the same pathogen isolated on two separate cultures or three of the five minor criteria: an elevated erythrocyte sedimentation rate (ESR) and C-reactive protein (CRP), elevated synovial leukocyte count, elevated percentage of polymorphonucleocytes (% PMNs), isolation of one organism in culture or greater than 5 PMNs per high-power field (400×) on histology. Electronic health records were reviewed to determine demographic data. MSIS criteria were retrospectively applied to each patient to ensure appropriately diagnosed infection.

Patient demographic data including age, sex, prior surgery and procedures, body mass index (BMI), and comorbidities, as well as American Society of Anesthesiologists (ASA) classification, were documented. The time periods were defined as interstage (after resection arthroplasty and prior to reimplantation), and post-reimplantation, with minimum of 1-year follow-up after reimplantation. Medical and surgical complications that occurred during the interstage and after the second-stage procedure were recorded and grouped together as dichotomous variables for analyses.

### 2.1 Study sample

The demographics of the study sample are presented in Table 1. Overall, there were 97 (48 %) females and 106 (52 %) males with a median age of 66 years (56 to 73 years). Nearly all patients were ASA III or IV, followed by ASA II and then V.

### 2.2 Statistical analysis

Study data were collected and managed using REDCap (Research Electronic Data Capture) and electronic data capture tools hosted locally (Harris et al., 2019, 2009). Redcap is a secure web-based software platform designed to support data capture for research studies. Frequency, proportion, measures of central tendency, and variance were calculated. Normality testing for all numeric data was performed. Independent-samples t tests or Wilcoxon rank sum tests were used for continuous normally or non-normally distributed data, respectively, for statistical comparisons. Chi-squared or Fisher's exact tests were used for categorical data to determine whether differences between independent variables were statistically significant. An a priori level of significance was defined as an α level of 0.05. All statistical analyses were conducted using SAS v. 9.4 (SAS Institute, Cary, North Carolina, USA).

**Table 1:** Demographics and comorbidities between patients who had a complication and did not have a complication following two-stage exchange arthroplasty. Note that DOS represents date of surgery.

		Complication		
	Overall (n=205)	No (n=132)	Yes (n=73)	P value
Age (in years) at index DOS, median (interquartile range (IQR))	65 (56, 73)	65 (57, 74)	63 (53, 71)	0.065
BMI at first stage, median (IQR)	28.8 (25.5, 33.7)	29 (25.8, 34)	28.2 (24.5, 33.1)	0.196
Sex, n (%)				
Female	99 (48.3 %)	63 (47.7 %)	36 (49.3 %)	
Male	106 (51.7 %)	69 (52.3 %)	37 (50.7 %)	0.828
Diabetes, n (%)				
No	162 (79.0 %)	103 (78.0 %)	59 (80.8 %)	
Yes	43 (21.0 %)	29 (22.0 %)	14 (19.2 %)	0.638
Renal failure, n (%)				
No	193 (94.1 %)	128 (97.0 %)	65 (89.0 %)	
Yes	12 (5.9 %)	4 (3.0 %)	8 (11.0 %)	0.029
Tobacco use, n (%) (missing for 3 patients)				
Never	101 (49.3 %)	68 (51.5 %)	33 (45.2 %)	
Former	72 (35.1 %)	47 (35.6 %)	25 (34.2 %)	
Current	29 (14.1 %)	14 (10.6 %)	15 (20.5 %)	0.162
ASA grade, n (%) (missing for 118 patients)				
II	10 (4.9 %)	3 (2.3 %)	7 (9.6 %)	
III	55 (26.8 %)	27 (20.5 %)	28 (38.4 %)	
IV	17 (8.3 %)	9 (6.8 %)	8 (11.0 %)	
V	4 (2.0 %)	4 (3.0 %)	0 (0 %)	0.136

## 3 Results

Overall, 73 of 205 patients (38 %) experienced at least one complication during the planned two-stage treatment for PJI. These 73 patients experienced a total of 114 complications over the course of the two-stage treatment, and 82 (72 %) of these complications required surgical treatment. The time of follow-up was 101 months (IQR 48, 169). The median time to complications was 103.5 months (IQR 46.5, 188.5). The overall mortality rate was 7.3 % (15 of 205). Five patients (2.4 %) died within the first postoperative year, and 10 patients died following the first postoperative year at an average of 2.5 years. There were no differences in age (p=0.065, independent-samples t test) or BMI (p=0.196, independent-samples t test) between patients who had post-reimplantation complications and those that did not. Table 2 lists the most common organisms causing infection during the index surgery. In 33 patients we were either unable to locate culture results or there was no growth noted at the time of resection on tissue cultures. We also did not find any difference in the infecting organism between those that did and did not have a complication. A total of 109 (53 %) patients were on chronic antibiotic suppression greater than 3 months (2 patients missing data point) and chronic suppression was significantly (p<0.0001; Fisher's exact test) more common among patients who experienced a complication (58 of 72 (79.5 %)) compared to patients who did not have a complication (51 of 141 (38.6 %)).

**Table 2:** Index surgery (first-stage) infecting organism.

	Overall (n=205)	No (n=132)	Yes (n=73)	P value
First-stage infecting organism, n (%)				0.060
MRSA (methicillin-resistant *Staphylococcus aureus*)	62 (30.2 %)	30 (22.7 %)	32 (43.8 %)	
MSSA (methacillin-sensitive *Staphylococcus aureus*)	40 (19.5 %)	26 (19.7 %)	14 (19.2 %)	
Coagulase negative staph	20 (9.8 %)	18 (13.6 %)	2 (2.7 %)	
Other strep species	16 (7.8 %)	10 (7.6 %)	6 (8.2 %)	
Enterococcus species	12 (5.9 %)	6 (4.5 %)	6 (8.2 %)	
*Escherichia coli*	5 (2.4 %)	2 (1.5 %)	3 (4.1 %)	
*Cutibacterium acnes*	4 (2.0 %)	3 (2.3 %)	1 (1.4 %)	
Polymicrobial	4 (2.0 %)	2 (1.5 %)	2 (2.7 %)	
Pseudomonas	4 (2.0 %)	2 (1.5 %)	2 (2.7 %)	
Corynebacterium	2 (1.0 %)	2 (1.5 %)	0 (0 %)	
Candida	2 (1.0 %)	1 (0.8 %)	1 (1.4 %)	
Klebsiella	1 (0.5 %)	0 (0 %)	1 (1.4 %)	

### 3.1 Interstage complications

During the interstage period, 27 % (56/205) of patients experienced a total of 75 total complications. The median time to complication between stages was 2.3 months (IQR 0.8, 4.0 months). Of the 75 complications, 24 were medical and 51 required surgery, which are presented in Table 3a. Ultimately, the interstage complication resulted in a failure to reimplant in 16 patients (16 of 205 (7.8 %)). Nineteen patients experienced 24 (24 of 75 (32 %)) medical complications, which did not require additional surgery, which occurred during the interstage period. Ten of those patients with a medical complication were unable to undergo the planned reimplantation surgery. Four patients died during the interstage period. Additionally, there were three thromboembolic events that required anticoagulation therapy.

**Table 3:** Interstage and post-reimplant complications.

**(a)** Interstage complications	
Medical	Patients, n
Medically compromised (failure to reimplant)	10
Death	4
Hematological (venous thromboembolic event)	3
Other	2
Surgical	Procedures, n
Aseptic reoperation	16
Septic reoperation	15
Wound complication requiring I&D	2
Spacer exchange (new infecting organism)	1
Spacer exchange (same infecting organism)	17
**(b)** Post-reimplant complications within 1 year	
Medical	Patients, n
Medically compromised (failure to reimplant)	1
Death	1
Wound complication	2
Low hemoglobin requiring transfusion	1
Instability	1
Surgical	Procedures, n
Aseptic reoperation	9
Septic reoperation (new infecting organism)	7
Septic reoperation (same infecting organism)	16

In addition to the 24 medical complications, 37 patients experienced a total of 51 complications that required additional surgery. To treat persistent PJI, 16 patients underwent an irrigation and debridement (I&D) with spacer exchange, 13 patients underwent an I&D without spacer exchange, and two patients underwent an I&D with spacer exchange to a static spacer. Additionally, two patients underwent an amputation (hip disarticulation), and one patient underwent a Girdlestone procedure to treat persistent PJI. Of the 35 complications related to persistent infection, one had growth of a new organism, while the others had persistence of the same organism present at time of initial surgery. Ninety-two percent of the spacers utilized at the time of initial resection were articulating spacers created with an intraoperative surgeon-directed mold. Only 8 % of the initial spacers were static non-articulating spacers owing to significant bone or soft tissue loss. There were eight articulating-spacer-related dislocation events that resulted in two spacer revisions and six closed reductions. Additionally, there were six periprosthetic fractures and 2 non-related surgical adverse events.

### 3.2 Post-reimplantation complications

Overall, 189 patients (92 %) underwent the second stage of a planned two-stage exchange arthroplasty. Within this group, 14 % (27 of 189 patients) of patients experienced at least one complication within 1 year of reimplantation. There was a total of 38 post-reimplantation complications, which are presented in Table 3b. Of these 38 complications, 6 (16 %) were medical, including 1 death, that did not require surgical treatment. There were 32 (84 %) complications that required surgery. In total, 16 (41 %) of the complications requiring surgical treatment were for recurrence of infection with the same organism, and 7 (18 %) of these recurrent infections involved a different organism. Of these 23 recurrent infections, 16 led to an I&D, 6 went on to a repeat two-stage procedure, and 1 underwent a resection arthroplasty. There were 9 surgical complications for instability, periprosthetic fracture, and heterotopic ossification. The interstage complication rate of 27 % (56 of 205 patients) was significantly (p=0.0003, McNemar's test) higher than the 14 % rate (27 of 189 patients) following the reimplantation surgery.

Table 4 lists the outcomes based on the MSIS definition of successful outcomes and guidelines for reporting (Fillingham et al., 2019). Of the 205 hips (203 patients), 122 (60 %) were successfully treated without antibiotic suppression or with antibiotic suppression 9 % (18 of 205) at 1 year and had no additional septic or aseptic revisions within 1 year of reimplantation. Thirty-two patients (15 %) retained their spacer and were not reimplanted at latest follow-up. Forty of the 205 (19.5 %) hips required surgical intervention to treat persistent PJI, and there was a 7.3 % mortality rate (15 of 203 patients).

**Table 4:** MSIS success.

		Overall
MSIS Success, n (%)		(n=205)
Tier 1: infection control, no chronic antibiotic suppression		122 (59.5 %)
Tier 2: infection control, on chronic antibiotic suppression		18 (8.8 %)
Tier 3: need for reoperation/revision and/or spacer retention		
	Tier 3A: aseptic revision >1 year from initiation of PJI treatment	3 (1.5 %)
	Tier 3B: septic revision >1 year from initiation of PJI treatment	2 (1.0 %)
	Tier 3C: aseptic revision <1 year from initiation of PJI treatment	4 (2.0 %)
	Tier 3D: septic revision <1 year from initiation of PJI treatment	3 (1.5 %)
	Tier 3E: amputation, resection, arthrodesis, Girdlestone procedure	6 (2.9 %)
	Tier 3F: retained spacer	32 (15.6 %)
Tier 4: death		
	Tier 4A: <1 year from initiation of treatment	5 (2.4 %)
	Tier 4B: >1 year from initiation of treatment	10 (4.9 %)

## 4 Discussion

The most common treatment for a chronically infected THA in the United States is a two-stage exchange arthroplasty (Parvizi and Della Valle, 2010). Success following this treatment ranges from 85 % successful in some studies to just over 60 % at the 1-year mark specifically (Wichern et al., 2020; Petis et al., 2019). This variability in success can be multifactorial with many risk factors having an impact on outcomes (Berend et al., 2013; Brown et al., 2018) Some of the reported success or failures, however, can also be due to how we define success and how we report the outcomes. The focus of our study was to look at outcomes and define our success over the whole course of treatment and not just following reimplantation, with specific emphasis on when during the treatment course complications occur, and we report our results using a standardized reporting measure established by the MSIS (Fillingham et al., 2019). Overall, while 92 % of patients that initiated the first stage of a two-stage exchange for chronic PJI of the hip underwent a reimplantation, only 68 % of our patients achieved tier 1 or 2 success and were deemed to be infection free at 1 year from reimplantation. Complications both during the interstage and post-reimplantation stages were high. Twenty-seven percent of patients experienced an interstage complication which included both medical and surgical complications, and 14 % of patients experienced at least one complication following reimplantation, most commonly persistence of infection in 59 % of those that had a complication.

Other studies have looked at complications related to PJI in THA with subsequent two-stage exchange arthroplasty. Cancienne et al. (2017) looked at patients who underwent two-stage reimplantation in PJI in THA in the Medicare database. They found that only 60.2 % of all patients underwent the second stage of a planned two-stage procedure. The in-hospital mortality was 6.5 %, 10.8 % required a repeat debridement for persistence of infection during the interstage phase, and 16.8 % had retained spacers indefinitely at the conclusion of this study. This is similar to the data that Gomez et al. (2015) reported on: they showed that out of 178 patients identified who underwent two-stage exchange for a chronically infected THA 2.8 % underwent a permanent resection arthroplasty and that 34 % had a retained spacer and were never reimplanted with a definitive prosthesis. Only 77 % of their study cohort was reimplanted, and they reported a 7.3 % 1-year mortality. Lange et al. (2016) showed a 63 % reimplantation rate of their two-stage revisions and a 92 % survivability at 1 year. They did note that their patients who underwent at least the first stage of the two-stage revision process were healthier than the ones who were not selected to do the two-stage revision process at all. In our study, complications were more likely to happen in the interstage period (27 %) than following reimplantation (14 %, p=0.003), most commonly persistence of infection, dislocation of an articulating spacer, and periprosthetic fracture. However, the complications and reoperations that occurred post second stage were not trivial. After the second stage, there were 27 patients that had a total of 39 complications. There were 23 persistent infections (59 % of the complications) that were classified as MSIS tier III or below, 5 dislocation events, and 1 permanent Girdlestone procedure. Overall, 32 patients (15 %) had a retained spacer either from the interstage or a recurrence of infection following reimplantation at latest follow-up.

The overall mortality of our study was 2.4 % (5 patients) at 1 year and 10 deaths total (4.9 %) at >1 year (2.5 years average) post-reimplantation; this is lower than what we reported in a similar study for total knee arthroplasties that underwent two-stage exchange which reported 3 % mortality in the interstage period, 4 % mortality within the year post-reimplantation, and an overall mortality of 18 % (Hartzler et al., 2020). This is notable as the patient population of both studies comes from the same catchment area with relatively similar comorbidity profiles and similar mean age (67 for Hartzler et al., 2020, versus 65 for this study). Our study showed that in the interstage period there was only one patient death (0.5 %). There was one additional patient death in the immediate post-operative period after reimplantation (0.5 %). These data are more optimistic than some of the studies in the literature that have reported higher mortality rates. Some of this may be due to the younger age on average of our patients as demonstrated in Lange et al. (2016); the average ages of the patients in that study were between 68 for reimplanted patients and 76 for non-reimplanted patients. Ibrahim et al. (2014) reported a 0.8 % 1-year mortality for two-stage exchanges in total hips but a 15.2 % mortality at the 5-year conclusion of their study. Cancienne et al. (2017) reported a 6 % in-hospital mortality but only a 60 % rate of reimplantation overall, which is a sobering reminder of the attrition that can occur between first and second stage. Gomez et al. (2015) reported on 178 patients with PJI in THA and saw a 7.3 % 1-year mortality rate. In the literature that combines THA and TKA (total knee arthroplasty) results, Barton et al. (2020) reported a combined mortality of 24 % when looking at PJI in both hips and knees. Zmistowski et al. (2013) reported a significantly higher mortality in the patient cohort undergoing revision for septic versus aseptic indications. They saw that at 90 d the mortality was 3.7 % versus 0.8 % for septic versus aseptic revisions, 10.6 % versus 2.0 % at 1 year, and 25.9 % versus 12.9 % at 5 years.

Our study was unable to demonstrate any differences between those that did and did not have a complication based on age, sex, or BMI. It is possible that this is related to the small overall numbers of patients in our study, as other studies have shown differences in outcomes based on patient comorbidities. Risk factors affecting outcomes and successful treatment have included age, BMI, sex, and medical comorbidities such as diabetes and renal failure (Eriksson and Lazarinis, 2020; Khan et al., 2019). In addition, patients on chronic antibiotic suppression appeared to have a higher rate of complication than those that were not on antibiotic suppression. Fifty-three percent of patients were on chronic antibiotic suppression for at least 3 months following reimplantation. This is contrary to many studies that demonstrate the protective effects of chronic antibiotic suppression following two-stage exchange arthroplasty (Petis et al., 2019; DeFrancesco et al., 2019). These findings may be for several reasons. During the period of this study, we did not have a standardized protocol for chronic antibiotic suppression, which therefore varied among surgeons. As a result, sicker patients with more virulent organisms, and those at higher risk for failure based on the surgeon's experience were most likely to be placed on chronic suppression. Therefore, from this study it is difficult to draw any conclusions about the role of chronic antibiotic suppression in treatment of PJI.

There has generally been a lack of agreement about what constitutes a successful treatment outcome for PJI. This makes it difficult to compare the outcomes between studies and ultimately between different treatment strategies. In order to bring standardization to the reporting of prosthetic infection-related outcomes, a work group from the MSIS published a series of definitions for successful infection management (Fillingham et al., 2019). This system stratifies outcomes of PJI from the initiation of treatment based on “success tiers”, ranging from successful reimplantation without chronic antibiotic suppression (tier 1) to death (tier 4). The system accounts for both septic and aseptic complications and their temporal relationship to the initiation of treatment for PJI. Our study demonstrated that while 92 % of patients ultimately had a reimplantation, only 68 % achieved a tier 1 or 2 outcome at 1 year defined as infection control without (tier 1) or with (tier 2) antibiotic suppression. This is similar to our study looking at complication in treatment of PJI for TKA. In that study by Hartzler et al. (2020), only 56 % of patient achieved tier 1 or 2 success at a minimum 1-year follow-up.

There are several limitations to this study, mainly its retrospective nature and the fact that the study period spanned more than 8 years during which time the techniques and management may have changed as with any retrospective study; there are also limitation with regards to accuracy of data collection and potential for selection bias. We attempted to minimize this by performing a manual chart review of every patient and excluding those that did not undergo at least the first stage of a two-stage exchange arthroplasty. However, it is possible that some were miscoded infection cases and therefore unable to be identified for the study. In addition, several surgeons were involved in the care of all of these patients which likely introduced treatment bias. Follow-up was only a minimum of 1 year; therefore, many of these patients may be at future risk for development of further septic or aseptic complications that have not been reported. Additionally, we did not include radiological or functional outcome measures. Using revision or reoperation (septic or aseptic) as an endpoint for success may overemphasize the success of patients with or without persistent problems who have not undergone further surgery by surgeon or patient choice.

Two-stage exchange arthroplasty remains that most common treatment for chronic PJI of the hip. Our study, and many of the more recent studies focusing on complications, would support the notion that the true success of two-stage exchange, when taking all factors into consideration, remains fraught with complications that truly affect the ultimate success. While success, defined as reimplantation of a prosthesis, may be high, the treatment course is associated with high rates of attrition and complications including recurrence of infection both during and after reimplantation.

The take-home message is as follows:

- The success of two-stage exchange arthroplasty for THA should include complications that occur during the interstage and post-reimplantation period.
- Although reimplantation rates may be high, complications including persistence of infection are common.

## Data Availability

Data were manually abstracted for this study, and the statistical analyses used are referenced in the Methods section. We cannot share the specific data analyzed due to patient healthcare privacy guidelines.

## Code availability

The REDCap or Research Electronic Data Capture code and software used in this study was purchased for usage by OrthoCarolina Research Institute. REDCap is a secure web application for building and managing online surveys and databases. While REDCap can be used to collect virtually any type of data in any environment (including compliance with 21 CFR Part 11, FISMA, HIPAA, and GDPR), it is specifically geared to support online and offline data capture for research studies and operations. The REDCap Consortium, a vast support network of collaborators, is composed of thousands of active institutional partners in over 100 countries who utilize and support their own individual REDCap systems. It can be found at (last access: 12 February 2021).
